# Pioneering and Interprofessional Pediatric Dentistry Programs Aimed at Reducing Oral Health Disparities

**DOI:** 10.3389/fpubh.2017.00207

**Published:** 2017-08-14

**Authors:** Francisco Ramos-Gomez, Hamida Askaryar, Cambria Garell, Jennifer Ogren

**Affiliations:** ^1^Section of Pediatric Dentistry, UCLA School of Dentistry, Los Angeles, CA, United States; ^2^Section of Pediatrics, UCLA School of Dentistry, Los Angeles, CA, United States; ^3^Department of Pediatrics, Mattel Children’s Hospital, David Geffen School of Medicine at UCLA, Los Angeles, CA, United States; ^4^Department of Neurobiology, University of California, Los Angeles (UCLA), Los Angeles, CA, United States

**Keywords:** children’s oral health, early childhood caries, preventive oral health care, oral health disparities, cultural factors, minimally invasive dentistry

## Abstract

Early Childhood Caries (ECC) is the most chronic childhood disease and more predominant in low-income and underserved children. Although easily transmitted, ECC is entirely preventable. Dr. Ramos-Gomez and his team at the University of California, Los Angeles put together an interprofessional curriculum where both medical and dental knowledge and practice is integrated to prepare dentists and primary care providers to more cost effectively address ECC and thereby reduce disparities in oral health. The curriculum, known as the Strategic Partnership for Interprofessional Collaborative Education in Pediatric Dentistry (SPICE-PD), consists of nine evidence-based training modules: applied statistics and research, community partners, interprofessional education/training, quality improvement, policy and advocacy, disease management/risk assessment, ethics/professionalism, cultural competency and children with special heath-care needs. SPICE aims to prepare pediatric dental residents and primary care providers to provide preventive, culturally competent, and minimally invasive oral care for underserved, low income, and special needs children. Additionally, the Infant Oral Care Program (IOCP), located at a local community health clinic, provides culturally sensitive preventive oral health care for children aged 0–5 years. The medical–dental integration model utilized at IOCP helps reduce oral health disparities by providing a systems-based and cost-effective approach to combat the burden of ECC. To track the progress of SPICE, a comprehensive evaluation framework has been designed, which aligns goals and objectives with program activities, desired outcomes, and measured indicators.

## Introduction

As stated in the Surgeon General’s 2000 report, Early Childhood Caries (ECC) is the most common chronic childhood disease, with a prevalence five times that of asthma (1). While it is highly infectious and transmitted easily from caregiver to child and from sibling to sibling, it is an entirely preventable disease (2–5). The key to prevention is early intervention and regular preventive dental care based on risk and disease management, but many families only seek dental care when problems occur. Remarkably, 80% of dental disease, including ECC, is concentrated in only 20–25% of the country’s children, who are primarily from low socioeconomic and/or minority backgrounds (5–7).

Early oral health interventions are critical for reducing the oral health disparities long suffered by the children of vulnerable and underserved communities (8, 9). Such interventions must be targeted in a socially, environmentally, and culturally appropriate manner, with a focus on early prevention, behavioral intervention, and collaborative involvement of dental and primary health-care providers.

Medical/dental integration is the goal of the Strategic Partnership for Interprofessional Collaborative Education in Pediatric Dentistry (SPICE-PD) residency curriculum at the University of California, Los Angeles (UCLA) School of Dentistry (10). This program is a continuation of the Community Health and Advocacy Training in Pediatric Dentistry, which spanned from 2010 to 2015. Both programs are funded by a grant from the Health Resources and Services Administration (HRSA), aim to augment and advance the training provided through the UCLA Pediatric Dentistry residency program to prepare dentists and primary care providers to meet the complex and comprehensive oral health needs of pediatric patients from underserved and high-need vulnerable populations more effectively.

The Infant Oral Care Program (IOCP), launched in 2010, is based on the assumption that low-income and minority caregivers visit venues like community clinics and Head Start/Early Head Start and Women, Infants, and Children (WIC) sites earlier and with more regularity than dental clinics. Therefore, these community program sites offer an ideal place where preventive dental services can most effectively be instituted. A low-cost medical–dental integrated preventive dental services model is instituted in these locations to help bridge the gap to better oral health for these underserved populations.

The objective of this paper is to showcase how the SPICE-PD curriculum and the IOCP through targeted and culturally appropriate means contribute to ameliorating oral health disparities and reducing the burden of oral disease.

## Rationale

Dental education has traditionally focused on surgical treatment of dental care, training oral health providers to rely primarily on restorative methods to maintain their patients’ oral health (11). As a result, many dental professionals are undertrained in aspects of preventive care, particularly when it comes to young children. In a 2015 study of 66 US and Canadian Dental Schools and in a study by the London Education and Training programme, it states that there is a lack of adequate science-based preventive dental education and preventive measures patient follow-up. For example, the former study concluded that it is evident that a great deficit is in reevaluation or outcomes assessment for preventive measure (12, 13).

Pediatricians and nurses also lack the training necessary to provide preventive oral health care that is effective. Both the American Academy of Pediatrics and US Preventative Services Task Force state that primary care providers should be knowledgeable on the management and prevention of dental caries (14, 15). Pediatric primary care providers are well positioned to help ameliorate the ECC disease burden with appropriate risk assessment, fluoride varnish application, and referral. Pediatric providers routinely see children for up to 10 well child checks by the time the child turns 2 years old, providing ample opportunity for risk assessment, fluoride application, and referral (16). Unfortunately, many pediatric clinicians lack the training necessary to provide this care. Indeed, a survey of medical schools found that 69% of schools had less than 5 h of oral health in their curriculum (17). Additionally, a survey of graduating pediatric residents found that over a third of pediatric residents who responded did not have any oral health training during their residency. Of those residents who did receive training, only 14% had any clinical time with a dentist (18). Curricula directly addressing this knowledge and training gap in pediatric oral health are needed to help reduce the burden of caries in children. This lack in adequate preventive oral health training results in two undesired and unnecessary outcomes.

First, children suffer needlessly through the pain of oral health disease. Clearly, this affects quality of life for the affected children, but can also impact school performance and overall health (19, 20). Families are also affected, missing valuable hours of work in order to deal with their children’s oral health issues. Second, reliance on restorative intervention to maintain oral health also costs our nation. A recent study by Bruen and colleagues on Medicaid expenditures for dental care suggests that, in 2011, there were approximately $450 million in additional Medicaid expenditures in Operating Room or Ambulatory Surgery Center-based surgical care for potentially preventable pediatric dental conditions, primarily related to ECC (21).

The goal of SPICE-PD is to create a framework for interprofessional training between pediatricians, nurses, and dentists that will directly target a known deficit in oral health training, and improve knowledge, confidence, and clinical performance in the prevention of ECC. The next generation of dentists and primary care providers needs to be ready to address the needs of those children most affected by the epidemic of ECC. To do this, it is imperative that we provide adequate community-based learning, incorporating community public health principles, and culturally appropriate preventive oral health-care practices into our pediatric curriculums.

SPICE also aims to enroll underrepresented minorities into their residency program in the hopes that once graduated they will serve and thereby help increase access to oral health care for vulnerable, underserved, or rural communities. Nationally, only about 8% of dental students and pediatric dental residents are Hispanic (22, 23), but 18% of CHAT/SPICE residents are Hispanic and 33% are from a disadvantaged background (2010–2017), far outpacing the national averages for those descriptors for dental students and pediatric dental residents (24).

## SPICE-PD: A New Education Strategy

The primary goal of the SPICE-PD program is to prepare pediatric dental residents to provide care for underserved and special needs groups and communities in the evolving field of dentistry. This HRSA funded program (2015–2020) includes nine training modules:

1. Applied statistics and research methods
2. Community partners
3. Interprofessional education (IPE)/training
4. Quality improvement
5. Policy and advocacy
6. Disease management/risk assessment
7. Ethics/professionalism
8. Cultural competency
9. NEW: children with special health-care needs [e.g., craniofacial abnormalities and autism spectrum disorders (ASD)].

**Figure 1:**
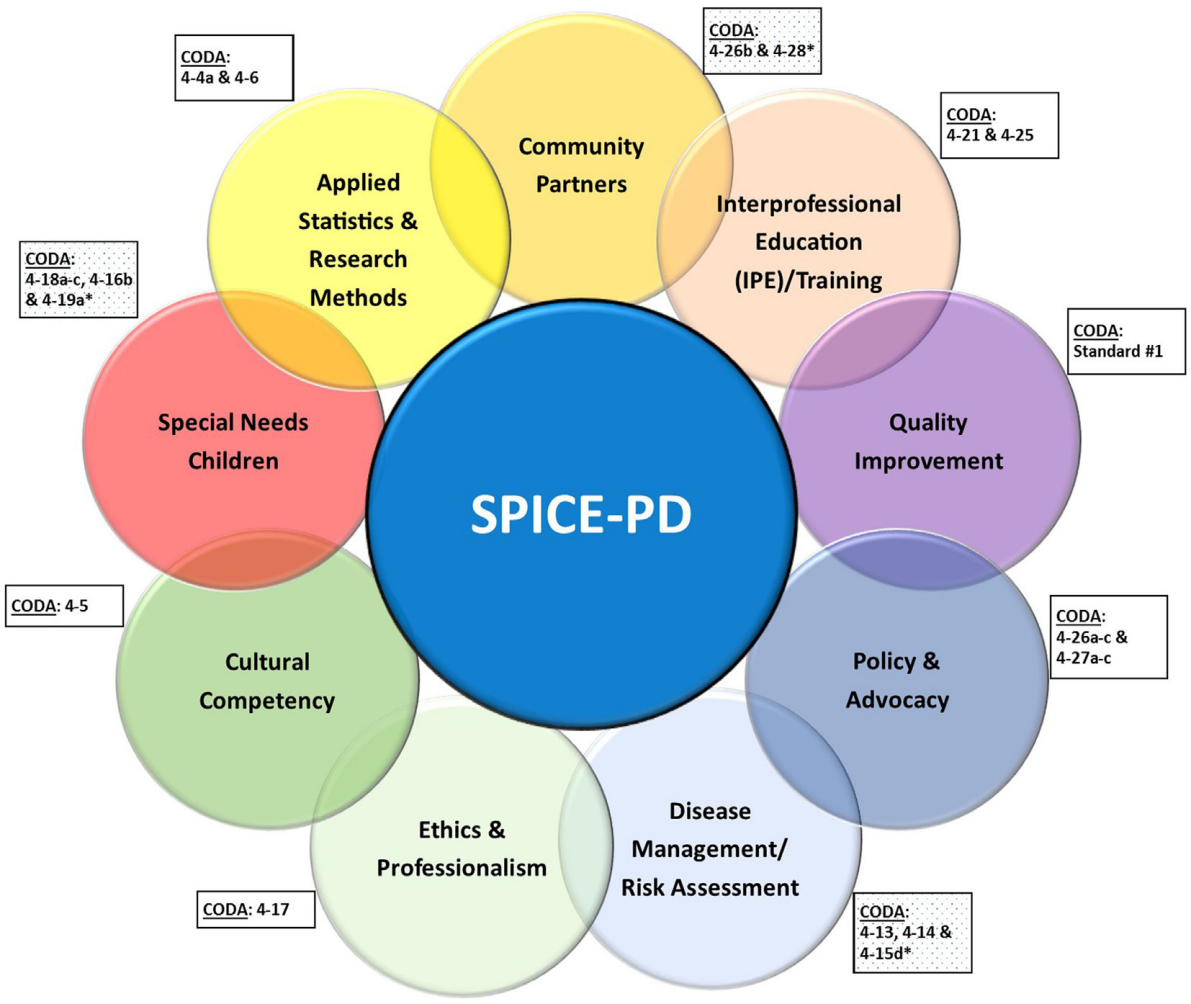
Strategic Partnership for Interprofessional Collaborative Education in Pediatric Dentistry (SPICE-PD) learning modules and their alignment with the Commission on Dental Accreditation (CODA) Standards for advanced education in pediatric dentistry.

Each module is designed to align with the Commission on Dental Accreditation (CODA) Accreditation Standards for advanced education in pediatric dentistry (Figure 1) (25). Each mandatory module on the average includes 21 h of didactics and/or hands-on/workshop training. The nine modules supplement the existing pediatric dental residency clinical curriculum.

Some topics, such as Applied Statistics and Research Methods, have long been part of the UCLA curriculum, but have been updated to reflect a public health emphasis, incorporating dental public health principles in addition to more traditional aspects of basic research methods and descriptive/inferential statistics. As a result, nearly 40% of the research topics chosen incorporate a public or community health emphasis, with more than 70% of the Class of 2016 choosing public health projects. Similarly, the Quality Improvement module represents a more traditional aspect of the curriculum, including the use of qualitative and quantitative methods to improve practice efficiency and safety, but also covers ways to improve the effectiveness of service delivery processes, particularly, those geared toward children from underserved and/or vulnerable populations.

Other topics aim to educate dentists on issues important to underserved, high risk, and vulnerable populations. One new module specifically addresses children with special health-care needs. The SPICE-PD curriculum focuses on medically complex special needs patients, as well as a clinical rotation at the Early Childhood Partial Hospitalization Program (ECPHP) at the Resnick Neuropsychiatric Hospital at UCLA, a program for children 2–6 years of age, diagnosed with ASDs and related comorbidities. The prevalence of ASD is disproportionately high in the greater Los Angeles area (26), with the highest distribution in Northern LA County, where the population is comprised primarily of low income families. Children with ASD are much more likely to have poor oral hygiene, decayed or missing teeth, and more likely to need restorative dental treatment (27). SPICE-PD residents train with ECPHP expert faculty to learn the most appropriate strategies for interacting with and providing care to this special population of patients.

Just as residents must become competent in strategies for interacting with children with ASD, Cultural Competency is vital when providing care in a multicultural community. In the Cultural Competency module, residents learn the importance of providing culturally and linguistically appropriate oral health care, while considering the impact of culture on attitudes, behavior, and oral health. This course helps residents identify and address health-care disparities and barriers to accessing oral health care, while developing a greater understanding of how to interact with a wide range of diverse patients and their families.

When addressing oral health in high risk groups, early intervention and strategic disease management are key. The Disease Management and Risk Assessment module stresses the importance of early assessment, diagnosis, and intervention as a means of oral disease prevention management. Early intervention and education are the most effective ways to prevent problems that traditional infectious-disease models fail to address, such as the epidemic of ECC (20, 28, 29). The module provides residents with a background in minimally invasive pediatric dentistry, individual oral health assessment, and treatment for pregnant women, infants, children, and caregivers. Central to this is the use of the Caries Management by Risk Assessment tool (CAMBRA) (4, 7, 30, 31), which provides a method of assessing caries risk in young children, thereby informing treatment plans, self-management goals, and recall schedules. In evidence-based minimally invasive dentistry, which includes use of CAMBRA, fluoride, sealants, and remineralization substances such as Casein Phosphapeptides, the patient/caregiver is encouraged to assume responsibility for the level of infection and is educated, instructed, and monitored in the proper control techniques. It is the patient/caregiver who has the disease, but it is the health professional’s responsibility to provide the patient the appropriate tools to overcome it.

Other modules are more directly aimed at achieving public health goals. CODA’s standards on Policy and Advocacy state that didactic instruction must cover the fundamentals of child advocacy, including issues pertaining to disparities in oral health-care delivery, such as access to care, as well as discussion of possible solutions (32). These topics are covered in depth throughout many of the SPICE-PD courses. But the new CODA guidelines go even further, highlighting the importance of exposing residents to various aspects of public health advocacy. The Policy and Advocacy module aims to prepare residents to be leaders in their field, advocating for children’s oral health and advising public health policy legislation at regional and national levels. Each year, our program sends all program year one pediatric dental residents to actively participate in the annual American Academy of Pediatric Dentistry advocacy days in Washington, DC (33), where they attend meetings with legislators to promote improved access to high-quality oral health care, particularly among vulnerable populations.

One of the most important aspects of advocating at a local level is through collaboration with local groups and community health programs. Community Partners module uses a systems-based approach to provide residents with a foundation for improving pediatric oral health within the context of their own community, laying a critical foundation for understanding the oral health-care delivery system. Specific topics include building relationships with local community organizations, public and private sector payers, and local policymakers. The course covers topics such as the social determinants of health and the problem with access to care, as well as an overview of Federal and State funded health programs, including Medicaid and CHIP, which provide dental care to underserved and financially disadvantaged populations. Pediatric residents must also complete an advanced practicum that involves teaching in community-based programs and teaching other health professionals.

The module on IPE provides pediatric dental residents with instruction needed to cross-train non-dental providers, such as pediatricians and nurses, on oral health disparities and dental development, as well as risk assessment, anticipatory guidance, and application of fluoride varnish. Residents have the opportunity to teach a class on children’s oral health to nursing students participating in the IPE program. The IPE course is an integral part of training the residents to work in interprofessional and multidisciplinary teams, which is key for early intervention. In addition to pediatric dental teaching experiences, all residents also cross train in a pediatric medicine rotation. These skill sets may also be utilized in the IOCP, where program year one pediatric dental residents work alongside pediatric medical residents as structured treatment teams. The experiences allows for bidirectional interprofessional exchange of knowledge and experience in caring for the pediatric patient from both the dental and medical perspectives.

The SPICE curriculum also includes training in preventive children’s oral health care for UCLA’s general dentistry residents. Far more, children in the US are seen by general dentists than are seen by pediatric dentists; therefore, the SPICE curriculum includes a 6-week interdisciplinary children’s oral health course for all general dentistry residents.

To track the success of the program, the SPICE-PD evaluation team has designed a comprehensive 5-year evaluation plan. At the center of this plan is the Evaluation Logic Model, which aligns SPICE-PD goals and objectives with program activities, desired outcomes, and measurable indicators. Table 1 describes the three focus areas (1. Program Development, 2. Learning, and 3. Professional Choices) and the corresponding evaluation key questions and data sources of the evaluation logic model. Each measure is defined with quantified metrics that will be tracked over time to enable measurement of the impact of the educational programs. As an important part of this evaluation logic model, the SPICE-PD team is working to bridge and integrate various electronic medical and dental records for patient tracking and quality improvement.

**Table 1:** Key Strategic Partnership for Interprofessional Collaborative Education in Pediatric Dentistry (SPICE-PD) key evaluation questions and data sources.

Focus area	Evaluation question	Data sources
Program development	What progress has the University of California, Los Angeles team made in establishing and improving SPICE-PD?	Program implementation tracking log Enrollment records Year-end survey of all SPICE-PD participants Faculty interviews Electronic dental/medical records

Learning	To what extent has SPICE-PD helped participants learn core competencies?	Year-end survey of all SPICE-PD participants Year-end SPICE exit exam Board exams

Professional choices	To what extent have SPICE-PD graduates applied core competencies and approaches to their practices?	Alumni survey
	To what extent have SPICE-PD graduates reported an increased commitment and preparedness to serve children from underserved and special needs populations? What proportion of graduates provides services to these children?	Alumni interviews

## The IOCP: Integrated Oral and Primary Health

The IOCP (9, 34) was founded in 2010 to address the dire needs of children in Los Angeles County. NHANES data from 1999 to 2004, showed that, compared to national norms, children in Los Angeles County were more likely to experience dental caries. In the primary dentition, nearly 40% of preschool children residing in LA County had dental caries compared to 28% of same age children in the US. Children residing in LA County had less favorable oral health than children in the US in 1999–2004 with ethnic minorities having the worst (6).

The program provides culturally sensitive preventive oral health care for children (aged 0–5 years) of low-income families in an integrated medical/dental setting. The objectives of the program include: giving patients access to early intervention in a community health setting, connecting caregivers early with a dental home, providing dental students and dental residents more in-depth pediatric experience, testing and implementing strategies for ECC prevention, and integrating pediatric dentistry with medical and nursing pediatric cohorts. By being located in a primary health-care facility with easy access and medical integration and by having the primary focus on prevention, IOCP helps break the barriers that contribute to disparities in oral health.

The IOCP rotation is part of the mandatory 1-week pediatric dental rotation for all dental students (87 dental students per year). In addition, each year, 12 dental students choose the IOCP 3-month rotation (total hours spent at IOCP in 3 months is 36 h) as a selective. Dental students, pediatric dental residents, international pediatric dental residents, medical residents, and advanced practitioner nursing students all participate side by side at IOCP rotations. The main goals of the IOCP rotation are for multidisciplinary trainees to gain proficiency in:

- Infant oral health exam techniques and procedures;
- Assessing caries risk using CAMBRA and clinical exam results;
- Examining, diagnosing, and treating the oral health needs of very young children;
- Working in a community-based clinical environment and understanding how to integrate infant oral care with the standard care delivered by pediatricians, nurse practitioners, and other providers.
- Understanding the barriers to accessing care for lower income families;
- Delivering perinatal and infant/toddler oral health education to caregivers.

The program has partnerships with Head Start/Early Head Start programs, WIC, and other community organization settings and is also easily replicable into those community settings. The advantage of IOCP is its limited overhead and start-up costs and potential to reduce health-care costs associated with oral disease. Unlike costly restorative/surgical care (21), the IOCP emphasizes education and prevention of dental diseases. This innovative approach helps reduce the burden of social disparities by providing preventive dental services and increased access to infants and toddlers from families of low socioeconomic status. This in turn prevents these families and children from losing valuable hours of work and school to attend to costly, risky, and time consuming restorative dental appointments. Preventive care at a typical IOCP visit includes the following six steps (35):

1. Caries risk assessment using CAMBRA,
2. Proper positioning using the “Knee-to-Knee” exam,
3. Toothbrush prophylaxis,
4. Clinical oral exam,
5. Fluoride varnish application,
6. Anticipatory guidance and self-management goals (using principles of motivational interviewing).

## Success of IOCP

An IOCP visit includes educating caregivers on early preventive oral health strategies and asking them to select two realistic self-management goals. Caregivers leave the clinic with a recall dental appointment that is based on the child’s risk assessment and is usually between 1 and 3 months. The IOCP clinic operates every Wednesdays year-round. From July 2010 to November 2016, IOCP served 950 patients’ aged 0–5 years for a total visit count of 2,572. Of the 950 children seen, full data are available on 908 children. Of these, 240 cases (26%) have been maintained with no decay, 38 cases (22% of all kids with white spot lesions) maintained at white spot lesions (and possibly have lesions arrested), and 39 cases (10%) were averted, which means that their white spot lesions were arrested and/or their caries were restored. Only 95 cases (10%) had worsening disease. The successful IOCP program rotation is one piece of the SPICE-PD curriculum that the UCLA pediatric dental residents and selected pediatric medical residents participate in during their residency programs. The goal is to augment and advance existing training to more effectively prepare residents to meet the growing oral health needs of children from underserved, minority, and high-need vulnerable populations.

## Conclusion

With the creation of IOCP and the addition of the SPICE curriculum, UCLA’s Division of Pediatric Dentistry has taken the unprecedented steps to battle the burden of ECC through community-based, evidence driven, and culturally sensitive ways. The comprehensive and progressive SPICE-PD curriculum focuses on teaching pediatric dental residents to effectively address the social determinants of oral health and thereby provide comprehensive care to the patient. At IOCP, dental students and residents learn and practice minimally invasive dentistry to address oral health disease at an early stage. Together, both projects have the potential to deliver better care, improve clinical outcomes, and reduce the overall cost of care and oral health disparities. Establishing an IPE program, in which minimally invasive dentistry is at the core of the curriculum, brings an innovative, systems approach to improve the oral health of the pediatric population through greater prevention and disease management by risk assessment, improvement in oral health literacy, cultural competency, and infrastructure development.

## Ethics Statement

UCLA IRB approval #16-000185.

## Author Contributions

All authors listed have made a substantial, direct, and intellectual contribution to the work and approved it for publication.

## Conflict of Interest Statement

The authors declare that the research was conducted in the absence of any commercial or financial relationships that could be construed as a potential conflict of interest.
